# Complete Genome Sequence of *Escherichia* Phage OSYSP

**DOI:** 10.1128/genomeA.00880-17

**Published:** 2017-10-19

**Authors:** Mustafa Yesil, En Huang, Xu Yang, Ahmed E. Yousef

**Affiliations:** a Department of Food Science and Technology, The Ohio State University, Columbus, Ohio, USA; b Department of Environmental and Occupational Health, University of Arkansas for Medical Sciences, Little Rock, Arkansas, USA; c Department of Microbiology, The Ohio State University, Columbus, Ohio, USA

## Abstract

Bacteriophage OSYSP is a new anti-*Escherichia coli* O157:H7 phage isolated from municipal wastewater in Ohio. OSYSP is potent against enterohemorrhagic *E. coli* and is a candidate biocontrol agent for food and therapeutic applications. In this paper, we present the important genetic features of this phage based on its complete genome sequence.

## GENOME ANNOUNCEMENT

Bacteriophages have received a great deal of attention as a natural biological control agent. Their easy applicability and host specificity are attractive to food processors for controlling foodborne pathogenic microorganisms. The U.S. Government continues to approve phage uses in food (1), which encouraged many researchers to pursue new bacteriophage-related studies. Previously, *Escherichia* phage OSYSP was shown to be effective against *E. coli* O157:H7 on experimentally contaminated fresh produce (2). In the current study, complete-genome sequencing was performed to determine the genetic characteristics of this promising phage and to assess its suitability and safety for use in food or therapeutic applications.

Extraction and purification of genomic DNA from pure *Escherichia* phage OSYSP suspension were carried out using the Norgen phage DNA isolation kit (Norgen Biotek Corp., Ontario, Canada), according to the manufacturer’s protocol. DNA library preparation and whole-genome sequencing were performed at the Department of Food Science at Pennsylvania State University. The DNA library was prepared using an Illumina Nextera DNA library preparation kit. Whole-phage DNA was sequenced in an Illumina MiSeq next-generation sequencing platform that generated 2 × 250-bp paired-end reads. *De novo* assembly of short raw reads was accomplished using the SPAdes 3.10.1 genome assembler software (3) that produced two large contigs. The gap between the contigs was successfully filled by PCR and Sanger sequencing.

Complete-genome sequencing of *Escherichia* phage OSYSP has revealed the genome size of 110,901 bp, with an average GC composition of 39.16%, which was determined by the Artemis software (4). Corrections to the coding sequences were also carried out using Artemis (4). Bacteriophage genes were predicted using GeneMarkS (5). Protein products and their functions were manually inspected by NCBI BLASTP using the nonredundant GenBank database (6). Genes encoding holin, endolysin, and host receptor binding proteins were found in the OSYSP genome, whereas lysogeny-associated genes were absent; these genetic results supported our previous findings of the lytic life cycle of the phage. Among 168 annotated genes, 100 encode new or hypothetical proteins. The phage genome contains 27 tRNAs, as predicted by Aragorn (7). The bacteriophage OSYSP genome has no genes encoding known antibiotic resistance, as determined using the Antibiotic Resistance Gene Database (8). An additional PCR was used to confirm the absence of Shiga toxin-encoding genes. The number of genes, tRNA, genome size, and GC content were found to be closely associated with *Escherichia* phages FFH1 (GenBank accession no. KJ190157), AKFV33 (accession no. HQ665011), and phiLLS (accession no. KY677846), which were also considered potential candidates as biological control agents (9–11). However, the assembled OSYSP phage genome showed a different arrangement from the aforementioned phage genomes; this observation was further confirmed by conventional PCR to rule out the possibility of false assembly of the OSYSP genome. Therefore, these results suggest that *Escherichia* phage OSYSP is a novel phage with the potential to be used as a biocontrol agent for medical or food safety applications.

### Accession number(s).

The complete genome sequence of *Escherichia* phage OSYSP has been deposited at the NCBI GenBank database and assigned the accession no. MF402939.
